# An Integrated Regulatory Network of mRNAs, microRNAs, and lncRNAs Involved in Nitrogen Metabolism of Moso Bamboo

**DOI:** 10.3389/fgene.2022.854346

**Published:** 2022-05-16

**Authors:** Tingting Yuan, Chenglei Zhu, Guangzhu Li, Yan Liu, Kebin Yang, Zhen Li, Xinzhang Song, Zhimin Gao

**Affiliations:** ^1^ Key Laboratory of National Forestry and Grassland Administration/Beijing for Bamboo and Rattan Science and Technology, Beijing, China; ^2^ International Center for Bamboo and Rattan, Institute of Gene Science and Industrialization for Bamboo and Rattan Resources, Beijing, China; ^3^ State Key Laboratory of Subtropical Silviculture, Zhejiang A and F University, Hangzhou, China

**Keywords:** moso bamboo, nitrogen metabolism, transcriptome, microRNA, long noncoding RNA

## Abstract

Nitrogen is a key macronutrient essential for plant growth and development, and its availability has a strong influence on biological processes. Nitrogen fertilizer has been widely applied in bamboo forests in recent decades; however, the mechanism of nitrogen metabolism in bamboo is not fully elucidated. Here, we characterized the morphological, physiological, and transcriptome changes of moso bamboo in response to different schemes for nitrogen addition to illuminate the regulation mechanism of nitrogen metabolism. The appropriate addition of nitrogen improved the chlorophyll content and Pn (net photosynthetic rate) of leaves, the nitrogen and ammonium contents of the seedling roots, the biomass of the whole seedling, the number of lateral roots, and the activity of enzymes involved in nitrogen metabolism in the roots. Based on the whole transcriptome data of the roots, a total of 8,632 differentially expressed mRNAs (DEGs) were identified under different nitrogen additions, such as 52 nitrate transporter genes, 6 nitrate reductase genes, 2 nitrite reductase genes, 2 glutamine synthase genes, 2 glutamate synthase genes (GOGAT), 3 glutamate dehydrogenase genes, and 431 TFs belonging to 23 families. Meanwhile, 123 differentially expressed miRNAs (DEMs) and 396 differentially expressed lncRNAs (DELs) were characterized as nitrogen responsive, respectively. Furthermore, 94 DEM-DEG pairs and 23 DEL-DEG pairs involved in nitrogen metabolism were identified. Finally, a predicted regulatory network of nitrogen metabolism was initially constructed, which included 17 nitrogen metabolic pathway genes, 15 TFs, 4 miRNAs, and 10 lncRNAs by conjoint analysis of DEGs, DEMs, and DELs and their regulatory relationships, which was supported by RNA-seq data and qPCR results. The lncRNA-miRNA-mRNA network provides new insights into the regulation mechanism of nitrogen metabolism in bamboo, which facilitates further genetic improvement for bamboo to adapt to the fluctuating nitrogen environment.

## Introduction

Nitrogen is a key macronutrient for plants and has a strong influence on crop development and productivity. There are different nitrogen forms available for plants, such as nitrate, ammonium, and a small amount of amino acid (Patterson et al., 2010). Nitrate is the major source of nitrogen due to its highly mobile and readily available features in the soil (Jin et al., 2015). Nitrate transporters (NRTs) are responsible for the uptake, transport of nitrate, and intracellular redistribution in plants, namely, NRT1, NRT2, and NRT3. Plants have evolved two different types of NRTs: high-affinity transport system (HATS) and low-affinity transport system (LATS), which are further divided into component and induction transport systems, respectively. After uptake by NRTs, a part of the nitrate is stored or assimilated in the roots while the rest is transported to the shoots (Xu et al., 2012). Several proteins or enzymes related to nitrogen metabolism play important roles in plant nitrogen assimilation, such as nitrate reductase (NR), nitrite reductase (NiR), glutamine synthase (GS), glutamate synthase (GOGAT), and glutamate dehydrogenase (GDH) (Gupta et al., 2019). Significant progress has been made in understanding nitrogen assimilation in plants (Wang et al., 2020; Li et al., 2020; Sanagi et al., 2021). For instance, the overexpression of nitrate transporter and nitrogen assimilation enzyme genes enhanced the ability of nitrogen uptake, with increased nitrate and ammonium contents, total nitrogen content, dry biomass, and yield in transgenic plants (Feng et al., 2011; Fang et al., 2012; Xia et al., 2015; Chen et al., 2016).

TFs have previously been identified to play important roles in nitrogen metabolism (Xu et al., 2016; Liu et al., 2017; Zhang et al., 2021). Many TFs have been reported to participate in the nitrogen response of *Arabidopsis thaliana*, and TCP20 plays a key role in the systemic signaling pathway that directs nitrate foraging by *Arabidopsis* roots (Brooks et al., 2019). The overexpression of *OsNLP1* promotes growth, nitrogen use efficiency, and grain yield in rice (Alfatih et al., 2020). In addition to protein-coding RNAs, emerging evidence has revealed that noncoding RNAs (ncRNAs) also play essential roles in nitrogen metabolism (Liu et al., 2019). Recent studies show that ncRNAs, such as microRNAs (miRNAs) and long noncoding RNAs (lncRNAs), function in many processes related to agricultural traits. Several large-scale investigations and functional studies of ncRNAs in plants suggested that they had significant regulatory effects on the physiological responses through regulating their targeted genes (Fischer et al., 2013), which led to improvements in some important agricultural traits such as productivity, male sterility, nutrient homeostasis, and floral organogenesis (Zhang et al., 2013; Fukuda et al., 2020). It has also been found that the changes in the nitrogen supply status alter the expressions of multiple miRNAs and lncRNAs in several plant species (Nguyen et al., 2015). Many miRNAs in plants were found to be involved in nitrogen metabolism (Lin et al., 2013; Yu et al., 2018), reprogramming root development (Vidal et al., 2010), and homeostasis of other nutrients (Paul et al., 2015). Although species-wide studies on plant miRNAs have been performed, few studies examining the involvement of lncRNAs in regulating nitrogen metabolism in plants have been reported (Chen et al., 2016; Fu et al., 2019; Zhai et al., 2020).

Moso bamboo (*Phyllostachys edulis*) is one of the representatives of woody bamboo, with 4.68 million hectares of forest area, accounting for about 73% of the total bamboo forest area in China (Li and Feng, 2019). Moso bamboo has a shorter growth cycle, strong regeneration ability, excellent mechanical strength, and high elasticity, which have made it a promising substitute for wood (Song et al., 2011). Moreover, bamboo shoot has become one of the indispensable foods to benefit from due to its low sugar and fat, and being rich in fiber characteristics (Hou et al., 2022). In addition, moso bamboo forests have high ecological value due to their strong carbon sequestration ability (Song et al., 2020). In production, a large amount of fertilizers, the majority of which are nitrogen fertilizers, are applied to obtain more bamboo shoots and culms, as well as higher ecological benefits. Recently, the molecular mechanism of nitrogen metabolism in moso bamboo has attracted much attention. A total of 13 *PeAMT*s, 27 *PeNPF*s, and 10 PeNLPs involved in nitrogen metabolism have been identified in moso bamboo (Li et al., 2021; Yuan et al., 2021a; Yuan, et al., 2021b). However, the internal regulation mechanism of nitrogen metabolism is still unclear. Based on the morphological and physiological changes of moso bamboo seedlings under different nitrate additions, we conducted multiple RNA-Seq analyses to find the TF and enzyme genes, as well as the lncRNAs and miRNAs involved in nitrogen metabolism. Finally, a predicted regulatory network of lncRNA-miRNA-mRNA was initially constructed. Our study provided new insights into the regulatory mechanism of nitrogen metabolism in bamboo.

## Materials and Methods

### Plant Material, Growth Conditions, and Nitrogen Treatment

We germinated and grew bamboo seeds in the substrate (peat:vermiculite = 7:3) in a greenhouse with a 16-h light (30°C)/8-h dark (28°C) photoperiod, 300 μmol m^−2^ s^−1^ photon density, and 60% humidity for 60 days. Next, 108 seedlings with a similar height (15 cm) and growth performance were selected and assigned to three groups, with 36 plants in each group. The seeds of these seedlings were removed, the roots were washed with deionized water, and then the plants were placed in a modified Kimura B solution (Wu et al., 2021) containing one of the following $NO_{3}^{-}$ concentrations: 0 mM (N0), 6 mM (N6), 12 mM (N12), 18 mM (N18), and 30 mM (N30). The nutrient solution for culture was renewed every 3 days. The treatments were maintained for 2 weeks until distinct morphological differences were observed among the treatments. The bamboo roots were harvested and part of them wrapped in tinfoil and immediately frozen in liquid nitrogen, and then stored at −80°C for further analysis.

### Determination of Physiological and Biochemical Indexes of Seedlings

To get the extracts, 0.2 g leaves were ground into powder and extracted with ethanol until the tissue turned white in darkness. The contents of chlorophyll (chlorophyll a and chlorophyll b) in the extracts were determined by a spectrophotometer (Ultrospec 3300 pro, Biochrom Ltd., Cambridge, England) at 470, 649, and 665 nm. Li-6400XT was used to measure the net photosynthetic rate (Pn) of the seedlings under different nitrogen additions. The red and blue lights of the cooperative determination system were used to determine the Pn from 9:00 to 11:00 a.m. The light intensity in the leaf chamber was 1,600 μmol m^−2^ s^−1^, and the CO_2_ concentration was 400 μmol m^−2^ s^−1^. The leaves were inserted into the leaf chamber, maintaining a normal growth situation as much as possible. The Pn of the seedlings was recorded when the data were stable. The biomass of the whole plant (dry weight) was determined.

The total nitrogen content was determined according to the Kjeldahl method (Ferrari, 1960). The content of nitrate, ammonium, NR (EC 1.7.99.4), NiR (EC 1.7.2.1), GS (EC 6.3.1.2), GOGAT (EC 1.4.7.1), and GDH (EC 1.4.1.2) activities were determined according to the instructions of the corresponding kits (G0440W, G0410W, G0402W, G0408W, G0401W, G0403W, and G0405W) produced by Suzhou Grace Biotechnology Co., Ltd. (Suzhou, China). Briefly, the samples that were weighed and extracted from the corresponding kit were added, followed by homogenization. Centrifugation was carried out, and the supernatant was taken for testing. The OD values were determined at 219, 570, 530, 540, 540, 340, and 450 nm. Then, the content of nitrate and ammonium and activity of the enzymes were calculated using the corresponding calculation formulas (Luo et al., 2013). All the measurements in this part were completed with three biological replicates.

### RNA Isolation, Library Construction, and RNA Sequencing

Total RNA was isolated using the TRIzol method for RNA-Seq. The integrity, purity, and concentration of the purified RNA were assessed with the Agilent 2100 Bio analyzer (Agilent, United States). Only high-quality RNA samples were used to construct the sequencing library. For the mRNA and lncRNA sequencing, 5.0 μg of total RNA was used to prepare rRNA (ribosomal RNA) removed strand-specific library using a TruSeq Stranded Total RNA Library Prep with the Ribo-Zero Plant Kit (Illumina, San Diego, CA, United States) according to the manufacturer’s instructions. There were three biological replicates per treatment, and a total of nine libraries were prepared. For small RNA sequencing, nine libraries were constructed with 3.0 μg of total RNA and the Truseq Small RNA Sample Prep Kit (Illumina, San Diego, CA, United States).

The denatured libraries were subjected to high-throughput parallel sequencing of both ends of the library using an Illumina HiSeq X™ Ten System sequencing platform. The quality of the raw data was evaluated using FastQC (version 0.10.1) with default settings. The clean data were separated using cutadapt (version 1.9), and the quality threshold was set to Q30, which removed the sequencing adapters and the primer sequence from the raw data to filter out low-quality data. phyllostachys_edulis.gigadb.HIC was used as the reference genome for sequence alignment and subsequent analysis (Zhao et al., 2018). The transcript level was quantified using Cufflinks (version 2.2.1), and the length of the transcript in the sample was normalized to Fragments Per Kilobase of exon model per Million mapped fragment (FPKM) values (Pertea et al., 2016).

### Identification, Functional Annotation, and Regulatory Relationships of Differentially Expressed mRNAs

To identify the DEGs between N0, N6, and N18 treatments, the expression level of each transcript was calculated according to the FPKM method. The DEGs were filtered with the criteria of a fold change (|FC|) ≥ 2 and false discovery rate (FDR) < 0.05 by DESeq2_EBSeq. The putative functions of the DEGs were determined according to nonredundant protein (NR) database. The Gene Ontology (GO) and Kyoto Encyclopedia of Genes and Genomes (KEGG) enrichment analyses were carried out using the OmicShare online platform (http://www.omicshare.com/tools/) [Accessed July 2021]. For the coexpression network analysis, the Weighted Gene Coexpression Network Analysis (WGCNA) package and the BambooNET database (http://bioinformatics.cau.edu.cn/bamboo/) [Accessed July 2021] (Ma et al., 2018) were used. The binding elements in the promoter were predicted by PlantRegMap (Tian et al., 2019), and the regulatory relationships between TFs, lncRNAs, miRNAs and nitrogen metabolism pathway genes were visually displayed using Cytoscape 3.7.2 (the same below).

### Identification of Differentially Expressed lncRNAs, Differentially Expressed miRNAs, and Their Targeted Genes

The prediction of candidate lncRNAs was performed by basic screening and potential coding ability screening. Briefly, the transcript sequences were assembled, annotated, and filtered based on their coding potential and length, in which the transcripts with potential coding ability were removed. Consequently, the remaining genes were regarded as candidate lncRNAs. To further investigate the potential roles of the lncRNAs, the expression level of each lncRNA was calculated according to the FPKM method. Differentially expressed lncRNAs (DELs) were extracted with an absolute value of |FC| ≥ 2 and FDR < 0.05 by DESeq2_EBSeq. The potential *cis-* and *trans*-targeted mRNAs of DELs were predicted according to their positions on the chromosome and expression correlation, respectively. For *cis* regulation, the lncRNAs were located within 10 kb upstream or downstream of their adjacent mRNA. For *trans* regulation, the expression level of the lncRNAs was opposite to that of their targeted mRNA, and the Pearson correlation between their expressions was ≥0.9 which was taken as the criteria for their targeted relationship.

The raw data were first quality controlled using the FASTX toolkit software (version 0.0.13, http://hannonlab.cshl.edu/fastx_toolkit/) to obtain clean small RNA reads by filtering out low-quality bases, sequencing adapters, reads shorter than 18 nt, and reads longer than 32 nt. The assembled unique sequences with clean reads were then BLAST searched against the Rfam database (version 12.1, http://rfam.sanger.ac.uk/) [Accessed January 2021] to remove non-miRNA sequences. The remaining reads were used to predict known miRNAs through a BLAST search of the miRbase (version 21.0, http://www.mirbase.org/) [Accessed March 2021], and novel miRNAs through analysis of the hairpin structure of the miRNA precursor with MIREAP (version 0.2) software. The expression level of each miRNA was calculated according to the transcripts per million (TPM) methods. DEMs were extracted with an absolute value of |FC| ≥ 2 and FDR < 0.05 by DESeq2_EBSeq. The targeted prediction of DEMs was performed with psRobot (version 1.01) (Zhang et al., 2014).

### Validation by Quantitative Polymerase Chain Reaction

To maintain the relative gene expression of the DEGs, DEMs, and DELs, qPCR was conducted as described by Yang et al. (2021). Briefly, cDNA was synthesized from 1.0 μg of total RNA with the HiScript^®^ II Q RT SuperMix (Vazyme, Cat#R223, China) for qPCR of mRNAs, the lnRcute lncRNA First-Strand cDNA Synthesis Kit (TIANGEN, Cat#KR202, China) for qPCR of lncRNAs, and the miRNA First-Strand cDNA Synthesis Kit (Vazyme, Cat#MR101, China) for qPCR of miRNAs. A qPCR assay was performed on a qTOWER 2.2 system (Analytik Jena, Germany) using the LightCycler 480 SYBR Green 1 Master kit (Roche, 04887352001), the lnRcute lncRNA qPCR Detection Kit (TIANGEN, Cat#FP402), and the miRNA Universal SYBR^®^ qPCR Master Mix (Vazyme, Cat#MQ101) following the manufacturers' instructions. The qPCR of each gene was carried out with particularly primers (Supplementary Table S5), and each experiment used three biological replicates. The 2^−ΔΔCT^ method was used to normalize and determine the RNA level relative to an internal reference gene *PeTIP41* (Fan et al., 2013) or *U6* snRNA (Ding et al., 2011).

### Statistical Analysis

For experimental variables, one-way analysis of variance (ANOVA) was used with nitrogen treatment as a factor, and differences between the means were considered significant when *p* < 0.05 and extremely significant when *p* < 0.01. The Ct values obtained from the qPCR were normalized and the relative fold changes in transcripts were calculated using the relative expression software tool. The heat map representing the expressions of genes was computed using the log_2_ (FPKM) by TBtools.

## Results

### Morphological and Physiological Changes of Moso Bamboo in Response to Nitrogen Availability

**FIGURE 1:**
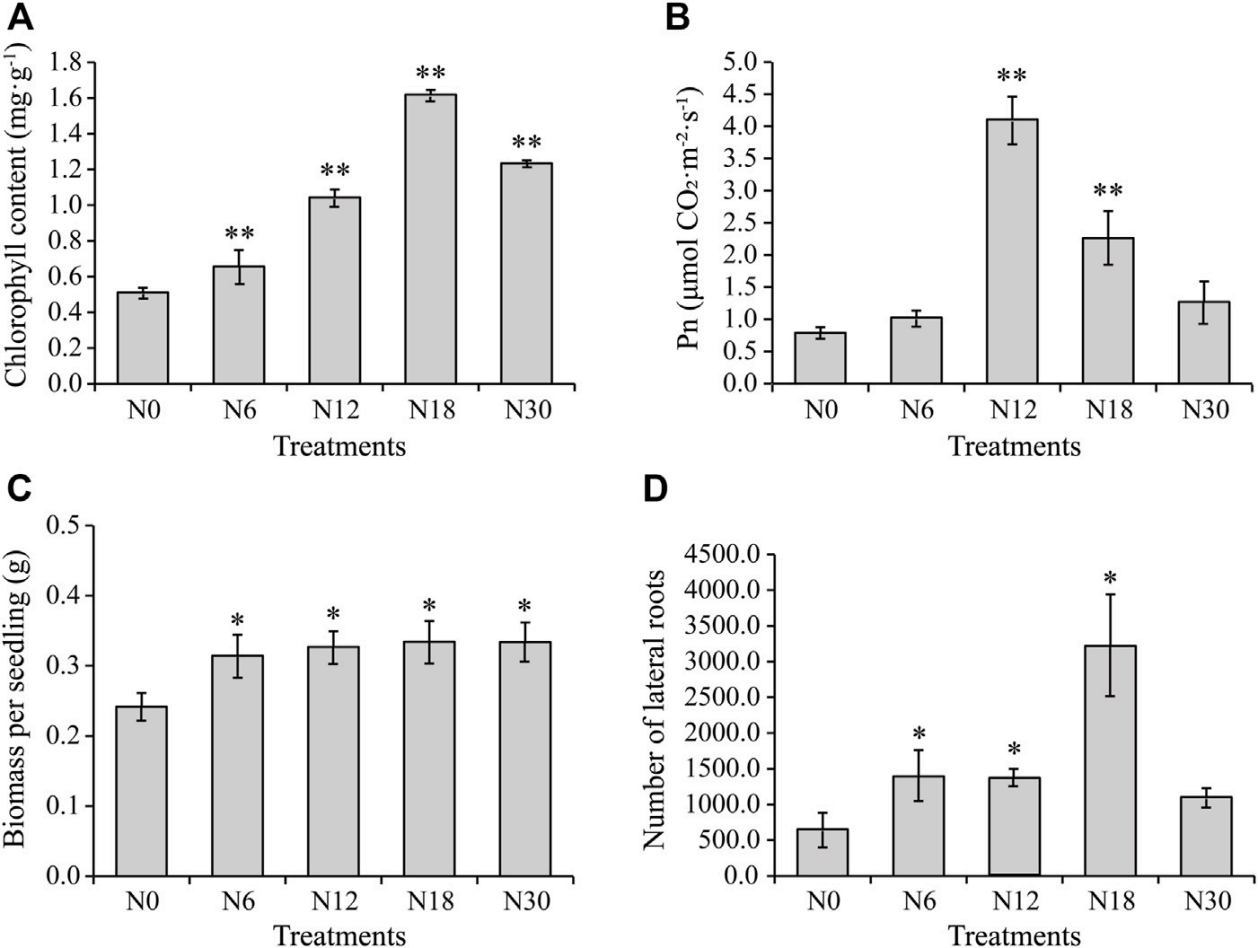
Physiological characteristics of moso bamboo seedlings under different nitrogen additions: **(A)** chlorophyll content of leaves, **(B)** Pn of leaves, **(C)** biomass per seedling, and **(D)** number of lateral roots from seedlings under different nitrogen additions. Data are means ± SD of three replicates (*n* = 3). Asterisks indicate the significant difference between N0 and other nitrogen additions (*means *p* < 0.05, ** means *p* < 0.01, the same below). RNA sequencing elucidated the DEGs in roots under different nitrogen additions.

The leaves of seedlings treated with N0 showed most severe chlorosis, while those treated with N18 and N30 showed similar normal color (Supplementary Figure S1A). The growth of roots was obviously promoted by nitrogen addition, except that N30 significantly inhibited it (Supplementary Figure S1B). Further measurement indicated that the chlorophyll content in the seedling leaves increased significantly under nitrogen addition when compared with that under N0, and it gradually increased with the increase of nitrate addition until it reached its maximum under N18, and then it slightly declined under N30 (Figure 1A). Moreover, Pn increased under all nitrogen additions with the maximum under N12, and there was a drop under N18 and N30 (Figure 1B), but it was still higher than under N0, which was similar to the changing trend of chlorophyll content. These results indicate that nitrogen addition within a certain concentration could increase the chlorophyll content and Pn of bamboo seedlings. Moreover, the total biomass of the seedlings further supported this inference; the increased biomass under nitrogen additions was not always increasing (Figure 1C). The number of lateral roots increased under nitrogen additions, which means nitrogen had a significant promoting effect on lateral roots (Figure 1D). The number of lateral roots were the most under N18 and decreased under N30 treatment. Therefore, seedlings under nitrogen additions of N0, N6, and N18 were selected for further studies.

Furthermore, the contents of total nitrogen, ammonium, and nitrate of the selected seedlings were also measured, and it showed that the contents of total nitrogen, i.e., ammonium under N6 and N18 was significantly higher than that under N0 (Supplementary Figures S2A, B), which indicated that nitrogen additions promoted nitrate uptake and assimilation in bamboo. However, the nitrate content in the roots under N6 and N18 was similar to that under N0 (Supplementary Figure S2C), suggesting that most of the nitrate absorbed by the roots may have been transported to other tissues. Besides, the activities of enzymes involved in nitrogen metabolism were measured, among which the activities of NR, NiR, and GOGAT were significantly increased and those of GS and GDH were significantly inhibited by nitrogen additions (Supplementary Figures S2D-H), indicating that nitrogen assimilation in the roots was induced by nitrogen additions in general.

To reveal the transcriptional regulation mechanisms that underlie the morphological and physiological changes of moso bamboo in response to nitrogen availability, genome-wide transcriptional analyses of RNA-seq data generated from the roots of seedlings under N0, N6, and N18 were conducted. A total of 191 million raw reads were obtained, with 22.6–42.2 million reads of each library. After sequence trimming, the number of clean reads per library still ranged from 21.9 to 41.0 million. About 17.7–36.1 million clean reads per library were mapped to the genome of moso bamboo, and the mapping ratio ranged from 71.2 to 87.9%. Finally, 49,292 genes were expressed in at least one sample out of the 59,481 genes detected totally.

**FIGURE 2:**
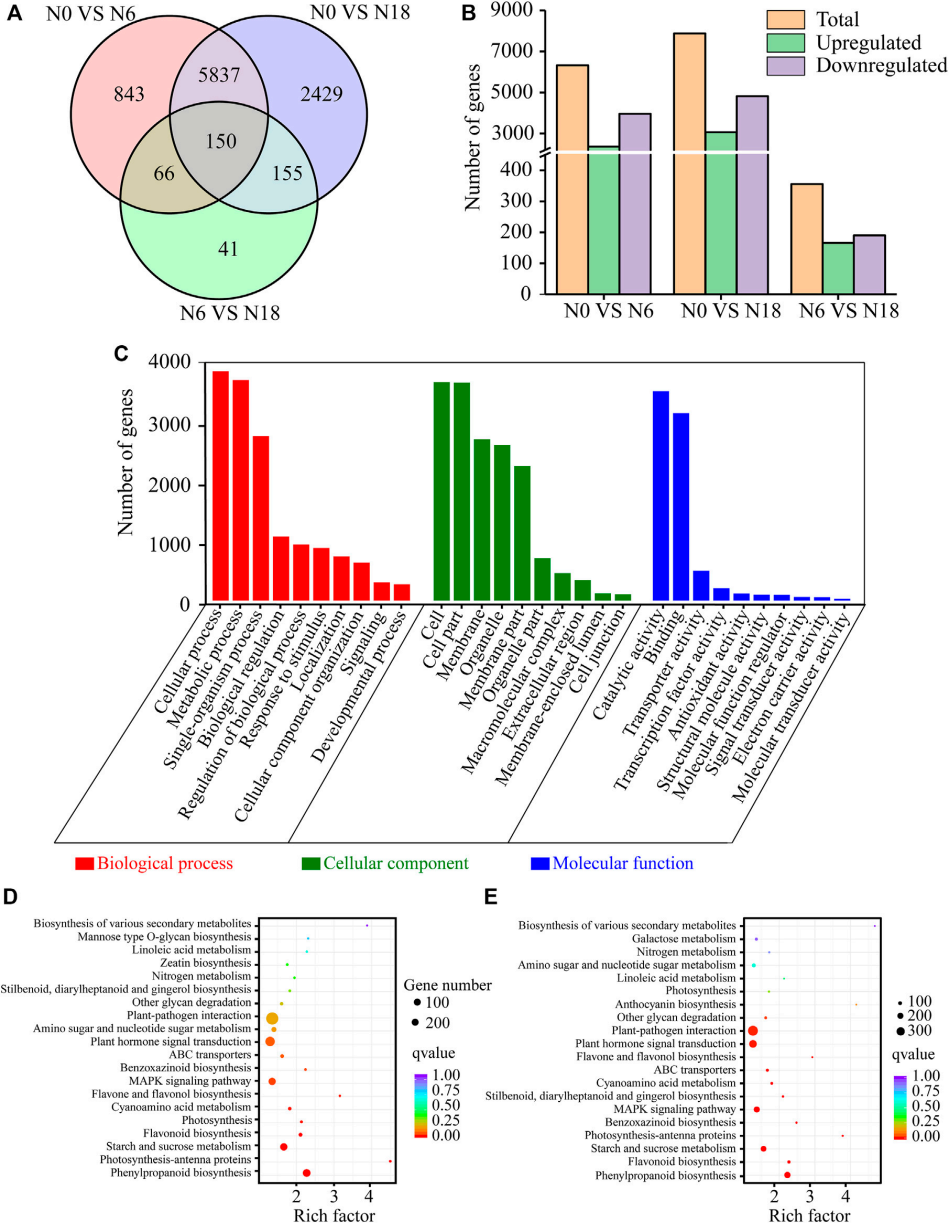
DEGs statistics and functional annotation: **(A)** Venn diagrams of DEGs; **(B)** number of upregulated and downregulated genes; **(C)** GO classification of DEGs; **(D)** KEGG enrichment of DEGs from N0 *vs*. N6; **(E)** KEGG enrichment of DEGs from N0 *vs*. N18.

A total of 8,632 genes were identified as DEGs, with 6,321 DEGs in N0 *vs*. N6, 7,884 DEGs in N0 *vs*. N18, and 356 DEGs in N6 *vs*. N18 (Figures 2A, B; Supplementary Table S1). Obviously, the number of DEGs in N0 *vs*. N18 and N0 *vs*. N6 was extremely far more than that in N6 *vs*. N18, indicating that nitrogen addition caused a series of changes in a wide range of biological processes involving a large number of genes in bamboo. Notably, the DEGs in N0 *vs*. N18 were also more than those in N0 *vs*. N6, which reminds us that the higher the nitrogen concentration, the greater the influence on bamboo. However, the concentration of nitrogen under N18 was three times of that under N6, while the number of DEGs in N0 *vs*. N18 was only 1,563 more than that in N0 *vs*. N6, indicating that the number of DEGs may not be totally attributed to the concentration of nitrate, but part of it was caused by the nitrogen present. These results further supported the nitrogen-free, nitrogen addition as well as nitrogen concentration caused by the different numbers of DEGs in bamboo.

### Functional Annotation and Classification of the Differentially Expressed mRNAs

To determine the biological functions, the DEGs were subjected to GO and KEGG pathway analyses. Out of 8,632 DEGs, 7,287 were annotated and divided into 53 major GO terms, namely, biological process (20 GO terms), cellular component (17 GO terms), and molecular function (16 GO terms). Of the biological processes, the terms with a high DEG number were metabolic process (GO:0008152), cellular process (GO:0009987), single-organism process (GO:0044699), biological regulation (GO:0065007), and response to stimulus (GO:0050896). Within the cellular component category, the most overrepresented terms were cell (GO:0005623), cell part (GO:0044464), membrane (GO:0016020), membrane part (GO:0044425), and organelle (GO:0043226). As for the molecular function, the most enriched terms were catalytic activity (GO:0003824), binding (GO:0005488), transporter activity (GO:0005215), nucleic acid–binding transcription factor activity (GO:0001071), and enzyme regulator activity (GO:0030234). Among the GO categories, the least frequent GO terms were cell killing (GO:0001906), extracellular matrix part (GO:0044420), and metallochaperone activity (GO:0016530) (Figure 2C).

KEGG enrichment analysis showed that the top 20 enriched pathways of the DEGs from N0 *vs.* N6, N0 *vs.* N18, and N6 *vs.* N18 were different from each other. Notably, the enriched pathways of N0 *vs.* N6 and N0 *vs.* N18 were similar, which were greatly different from those of N6 *vs*. N18. In comparisons of N0 *vs*. N6 and N0 *vs*. N18, the most enriched pathway was phenylpropanoid biosynthesis (ko00940), and nitrogen metabolism (ko00910) was also significantly enriched in both comparisons. As for the comparison of N6 vs. N18, starch and sucrose metabolism (ko00500) was the top enriched pathway, and phenylpropanoid biosynthesis (ko00940) was ranked last, while nitrogen metabolism (ko00910) was not enriched in the top 20 pathways (Supplementary Figure S3). These results indicated that similar changes of genes involved in phenylpropanoid biosynthesis and nitrogen metabolism occurred in bamboo under nitrogen addition. However, there were differences between N0 *vs*. N6 and N0 *vs*. N18, such as starch and sucrose metabolism were strengthened in N0 *vs*. N18, while phenylpropanoid biosynthesis and nitrogen metabolism were weakened in N0 *vs*. N6, indicating that nitrogen addition might change the flow direction of nitrogen and carbon, resulting in the change of metabolic pathways in bamboo (Figures 2D, E).

### Expression Patterns of Transporter and Enzyme Genes Involved in Nitrogen Metabolism

**FIGURE 3:**
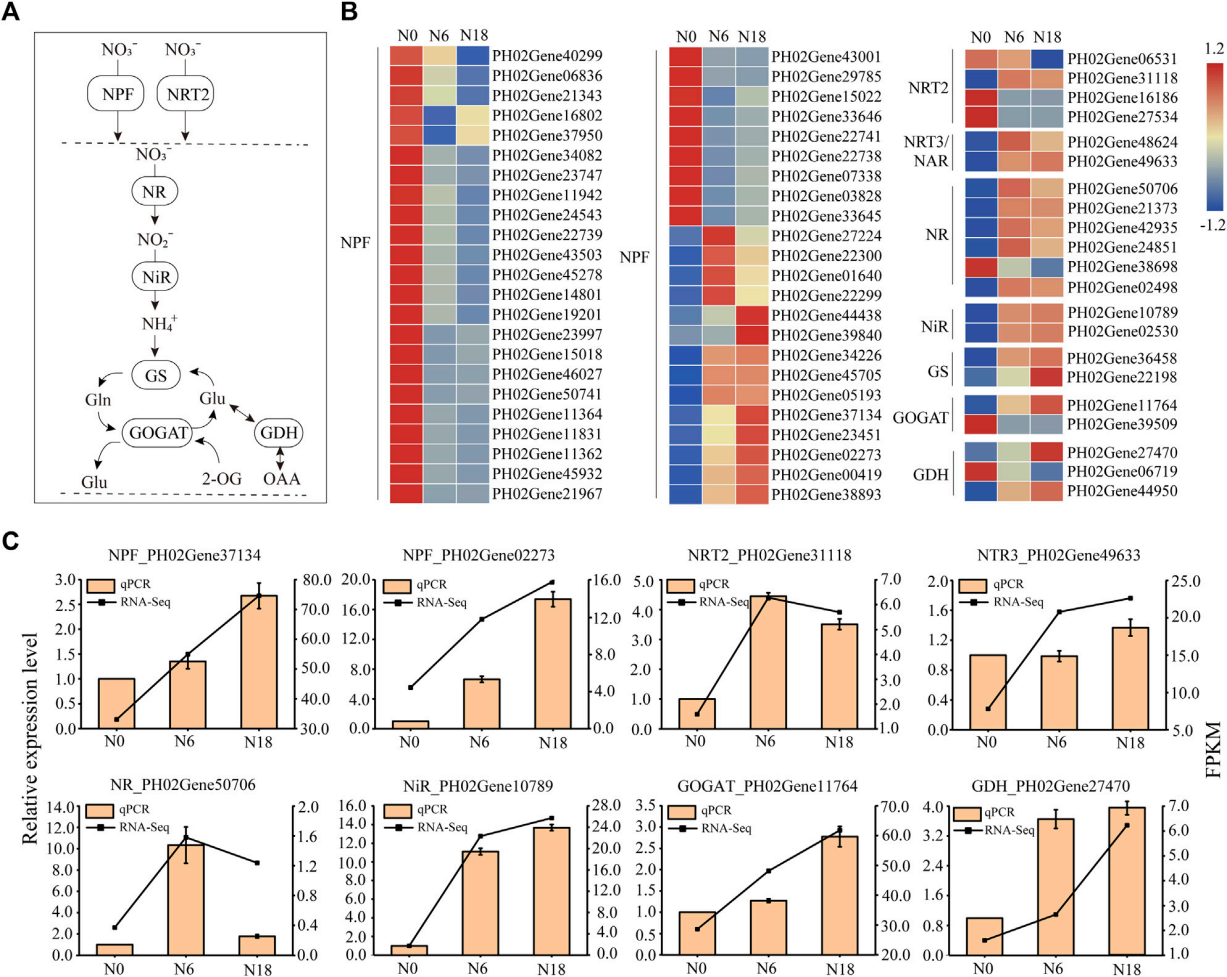
Expression profiles of genes involved in nitrogen metabolism of bamboo roots: **(A)** outline of nitrate uptake and assimilation; **(B)** heat map visualization of DEG expression profiles; **(C)** expression analysis of eight DEGs belonging to the NPF, NRT2, NRT3, NR, NiR, GOGAT, and GDH families. Expression profiles of TFs that participated in nitrogen metabolism.

The expression patterns of transporter and enzyme genes involved in nitrate uptake, transport, and assimilation were analyzed. Totally, 67 DEGs of 8 families, namely, NPF, NRT2, NRT3/NAR2, NR, NiR, GS, GOGAT, and GDH, were detected (Figures 3A, B). Among these families, NPF contained the most DEGs, in which the expressions of 32 members were inhibited and 10 members were induced by nitrogen additions, while 4 members were first induced under N6 and inhibited under N18. The diversity of expression patterns indicated that *PeNPF*s function widely in nitrogen uptake and transport, which is consistent with those NPF members in *Arabidopsis* and rice (Hu et al., 2014; Wang et al., 2018). In addition, three members of *PeNRT2* were induced under N0 and inhibited under N6 and N18, while another member of *PeNRT2* was induced under N6 and N18, which was similar to those members of *NRT3*/*NAR2*. Most DEGs involved in nitrogen assimilation were induced by nitrogen additions, and their expressions increased with the nitrogen concentration, such as the members of the NiR and GS families. Three DEGs belonging to different nitrogen assimilation families were induced under N0 and suppressed under N6 and N18. These results were further validated by eight randomly selected DEGs using qPCR, which showed a consistent trend with the transcriptome (Figure 3C).

**FIGURE 4:**
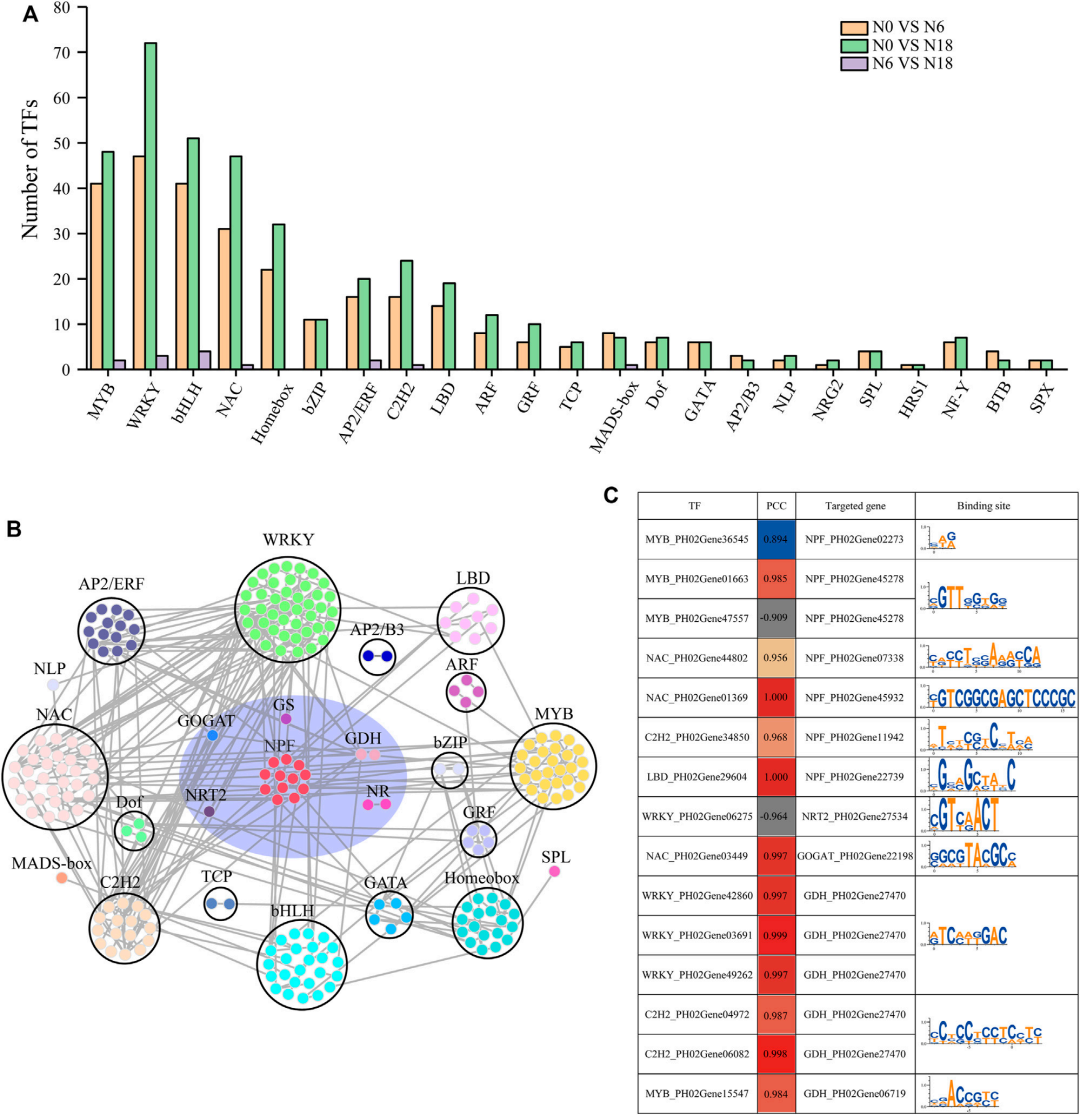
TFs identified from DEGs and their targeted genes involved in nitrogen metabolism: **(A)** statistics of TFs; **(B)** coexpression network of TFs and nitrogen metabolism genes based on WGCNA and BambooNET; **(C)** PCC of TFs and their targeted genes involved in nitrogen metabolism. miRNA sequencing uncovered the DEMs in roots under different nitrogen additions.

TFs play important roles in regulating plant growth and adapting to the environment. According to previous reports, more than 20 TF families are involved in nitrogen-mediated biological processes (Gaudinier et al., 2018; Varala et al., 2018). In this study, 431 TFs belonging to 23 families were identified from the DEGs, and the top 10 families with the most members were WRKY (74);, basic helix–loop–helix (bHLH) (60); MYB (51); NAM, ATAF, and CUC (NAC) (51); ERF/AP2 (24); homeobox (33); C2H2 (24); LBD (20); bZIP (13); and ARF (12). Besides, other families with a few members of DEGs were also concerned due to their significant differences in comparisons of N0 *vs*. N6, N0 *vs*. N18, and N6 *vs*. N18, such as BTB (4), NLP (3), AP2/B3 (3), NRG2 (2), SPX (2), and HRS1 (1) (Figure 4A).

To find the transcriptional regulatory relationship, the co-expressed which were differently expressed TFs, and the transporter and enzyme genes involved in nitrogen metabolism were identified. In total, 246 gene pairs from 18 TF families and 6 transporter and enzyme families were identified (Figure 4B), among which 34 pairs of TF-nitrogen metabolism genes were found with a Pearson correlation coefficient (|PCC|) > 0.8 and the *p* < 0.05. Furthermore, we analyzed the binding elements in the promoters of the nitrogen metabolic pathway genes in the 34 pairs. Finally, 15 pairs were identified as regulatory pairs, containing 10 nitrogen metabolic pathway genes and 15 TFs. The conserved regulatory elements targeted by TFs in the promoters of the nitrogen metabolic pathway genes are provided in Figure 4C.

**FIGURE 5:**
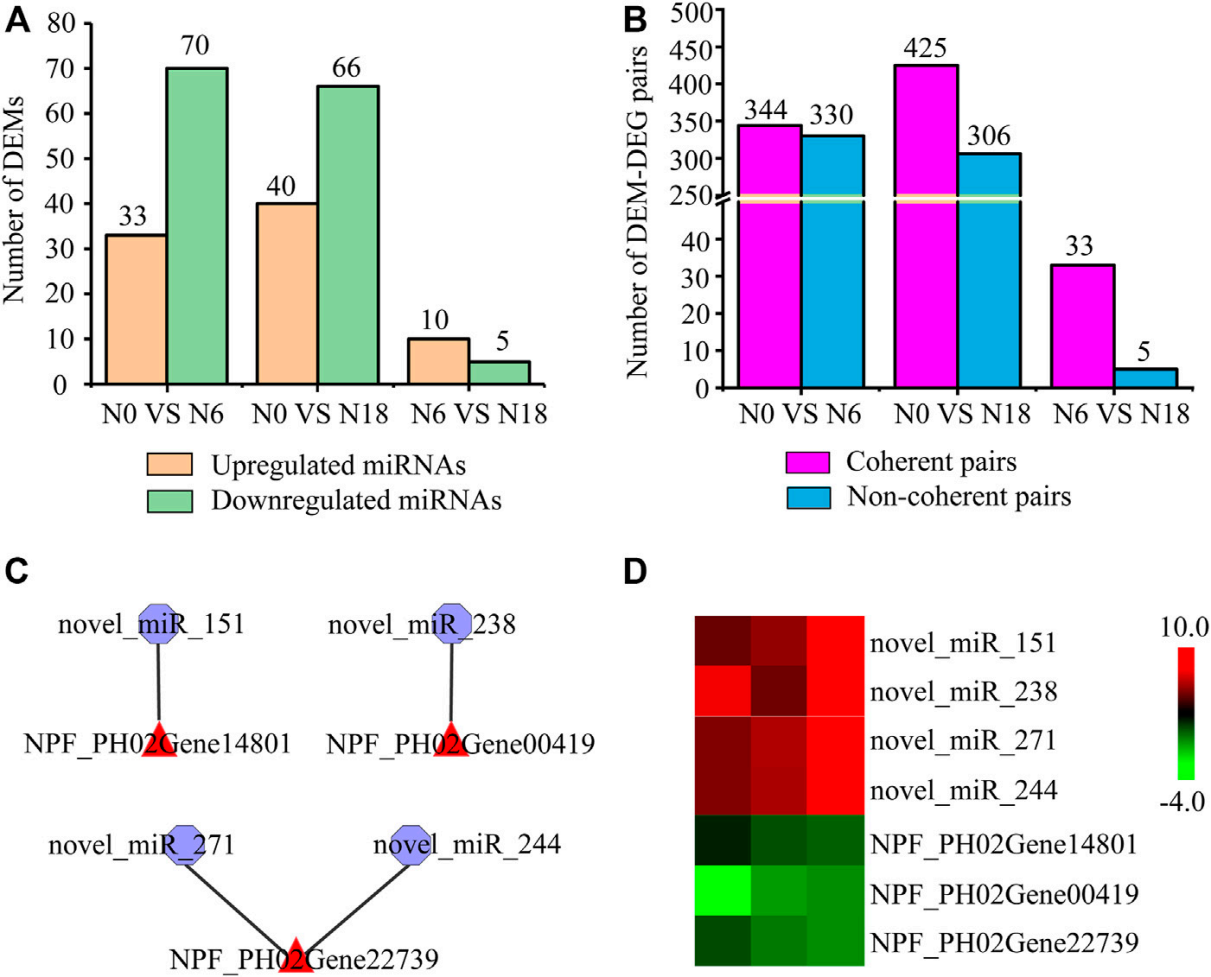
Analysis of DEMs and their targeted genes: **(A)** statistics of DEMs; **(B)** statistics of DEM-DEG pairs with similar or opposite expression patterns; **(C)** DEM-DEG pairs involved in nitrogen metabolism; **(D)** expression analysis of DEM-DEG pairs using transcriptome data. Heat map colors varied from green to red, representing low to high gene expression.

A total of 43,326,868 raw reads were generated from 9 small RNA root libraries. After removing the reads of low-quality contaminated adapter sequences, the reads with a base length between 18 and 30 nt were further analyzed as typical miRNAs. A total of 383 miRNAs were identified, namely, 62 known miRNAs and 321 newly predicted ones. To explore the expression level changes and potential regulatory roles of these miRNAs, we calculated the expression levels of all the miRNAs and analyzed the DEMs by pairwise comparisons. Among the DEMs identified in moso bamboo, 33, 40, and 10 were upregulated, while 70, 66, and 5 were downregulated in three comparisons (Figure 5A; Supplementary Table S2). The interaction of miRNA-mRNA can be either coherent with the opposite expression tendency or noncoherent with a similar expression tendency (Garg et al., 2019). Furthermore, 344, 425, and 33 coherent pairs, and 330, 306, and 5 noncoherent pairs were identified in N0 *vs*. N6, N0 *vs*. N18, and N0 *vs*. N18, respectively (Figure 5B).

Moreover, among these different expression patterns of DEM-DEG coherent pairs, we focused on those miRNAs which were targeted genes involved in nitrogen metabolism. Totally, 94 DEM-DEG pairs (involved in nitrogen metabolism) were identified, among which 4 were DEM-nitrogen metabolic pathway gene pairs and 90 were DEM-TF pairs. The above four pairs comprised three *NPF* genes (PH02Gene00419, PH02Gene14801, and PH02Gene22739), which were targeted by *novel_miR_238*, *novel_miR_151*, *novel_miR_244*, and *novel_miR_271*, respectively (Figure 5C), and the opposite tendency of transcriptome expression patterns further supported the regulatory relationship between them (Figure 5D). The 90 pairs included 22 differentially expressed TFs from GRF, ARF, C2H2, NAC, AP2/ERF, bZIP, homeobox, MADS-box, NF-Y, SPL, and WRKY, which were targeted by 29 DEMs (Supplementary Figure S4), such as *GRF*_PH02Gene03385 was targeted by *novel_miR_70*, *novel_miR_262*, *novel_miR_7*, *novel_miR_106*, *novel_miR_56*, *novel_miR_247*, *novel_miR_250*, and *novel_miR_162*, while *C2H2*_PH02Gene43070 and *MADS-box*_PH02Gene50032 were targeted by *novel_miR_223* and *novel_miR_333*, respectively.

### lncRNAs Sequencing Analysis Uncovered the Differentially Expressed lncRNAs in Roots Under Different Nitrogen Additions

**FIGURE 6:**
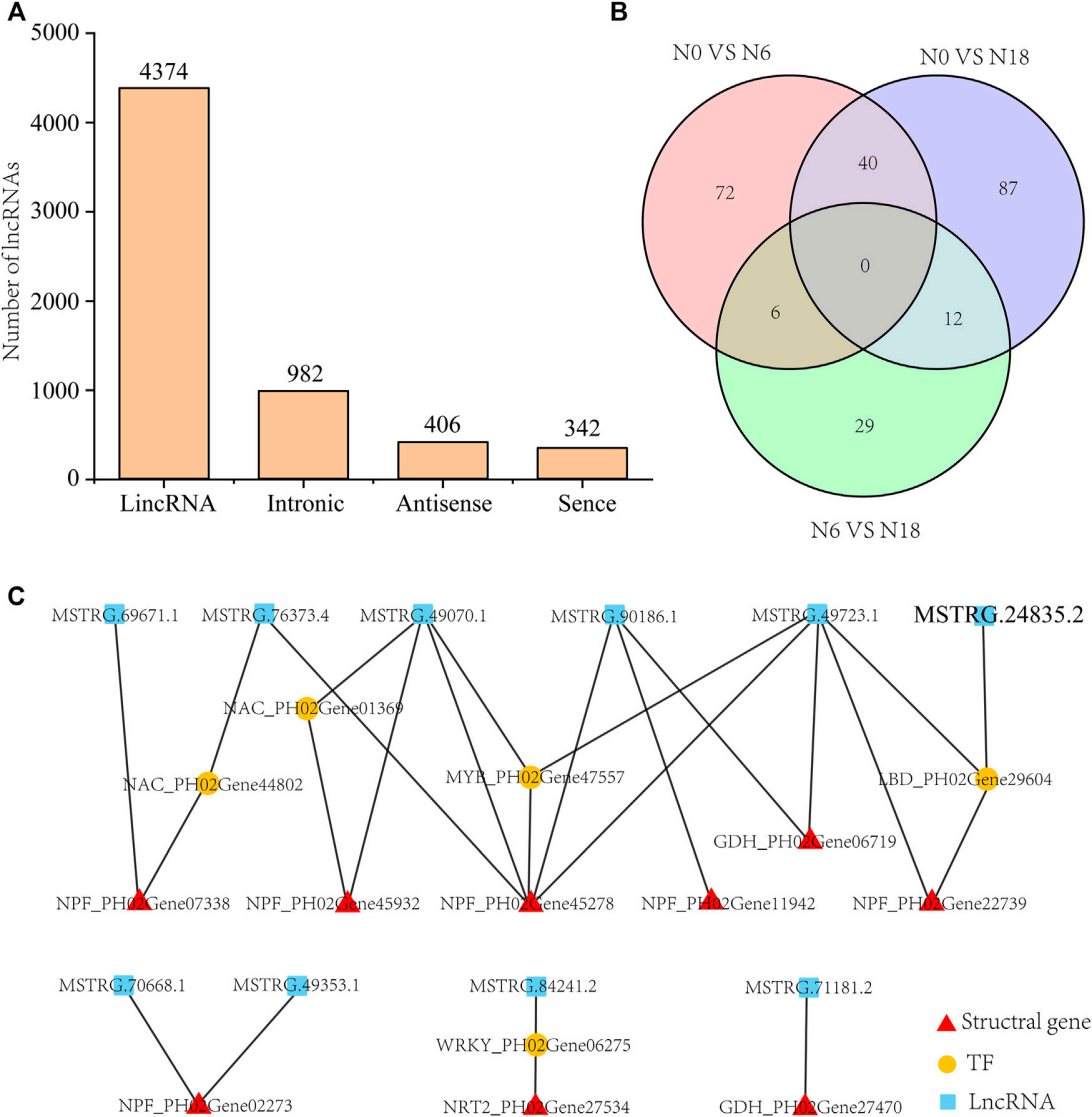
Analysis of DELs and their targeted genes: **(A)** category and number statistics of lncRNAs; **(B)** Venn diagram of DELs from N0 *vs*. N6, N0 *vs*. N18, and N6 *vs*. N18; **(C)** DEL-DEG pairs involved in nitrogen metabolism.

Altogether, 6,104 novel lncRNAs were identified from the 9 cDNA libraries, among which the numbers of lincRNAs, intronic, antisense, and sense were 4,374 (71.7%), 982 (16.1%), 406 (6.7%), and 342 (5.6%), respectively (Figure 6A). In the predicted lncRNAs, 396 DELs were identified (Supplementary Table S3), namely, 118 DELs (43 upregulated and 75 downregulated) of N0 *vs*. N6, 139 DELs (62 upregulated and 77 downregulated) of N0 *vs*. N18, and 47 DELs (26 upregulated and 21 downregulated) of N6 *vs*. N18 (Figure 6B). To further investigate the potential roles of DELs, we first identified the DELs that regulated their targeted genes in *cis* or *trans*. Totally, 237,017 pairs, 247,214 pairs, and 9,315 pairs of DEL-targeted genes were identified, among which 127,314 pairs, 146,008 pairs, and 938 pairs were identified as DEL-DEGs in N0 *vs*. N6, N0 *vs*. N18, and N6 *vs*. N18, respectively.

LncRNAs participated in nitrogen metabolism by directly targeting the nitrogen metabolism pathway genes or indirectly by binding to the TFs that targeted them. Twenty DEL-DEG pairs were identified, which included DEGs of transporters, enzyme genes, and TFs (Figure 6C). Eight lncRNAs directly targeted nitrogen metabolic pathway genes, i.e., one-to-one, many-to-one, and one-to-many, such as *MSTRG.71181.2* targeted *GDH*_PH02Gene27470, and *MSTRG.90186.1* targeted *NPF*_PH02Gene45278, *NPF*_PH02Gene11942, and *GDH*_PH02Gene06719, while *MSTRG.76373.4*, *MSTRG.49070.1*, and *MSTRG.49723.1* targeted the same *NPF*_PH02Gene45278. Four lncRNAs regulated nitrogen metabolic pathway genes by binding to their targeted TFs, such as NAC_PH02Gene44802, NAC_PH02Gene01369, MYB_PH02Gene47557, LBD_PH02Gene29604, and WRKY_PH02Gene06275. Finally, we identified 10 lncRNAs those which formed 20 gene pairs with the nitrogen metabolic pathway genes or TFs, participating in the response to different nitrogen additions (Figure 6C).

### Integration and Validation of the Regulatory Network of Nitrogen Metabolism in Moso Bamboo

**FIGURE 7:**
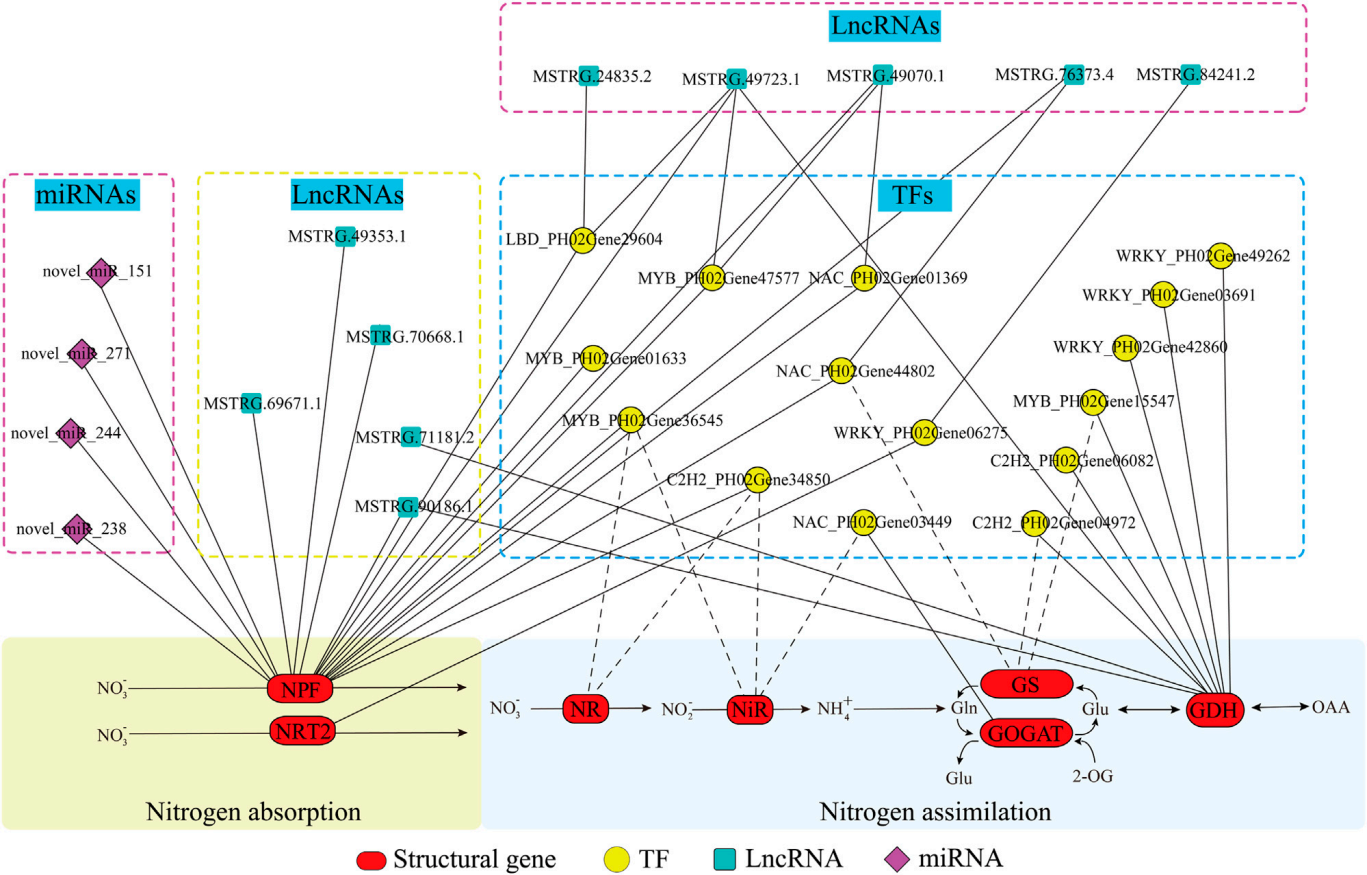
Predicted regulatory network of nitrogen metabolism in moso bamboo. Structural genes, TFs, miRNAs, and lncRNAs are marked with red ovals, yellow circles, blue blocks, and purple rhombuses, respectively. The solid lines and dotted lines indicate the potential regulated relationship between the two elements supported by experiments and only prediction, respectively.

**FIGURE 8:**
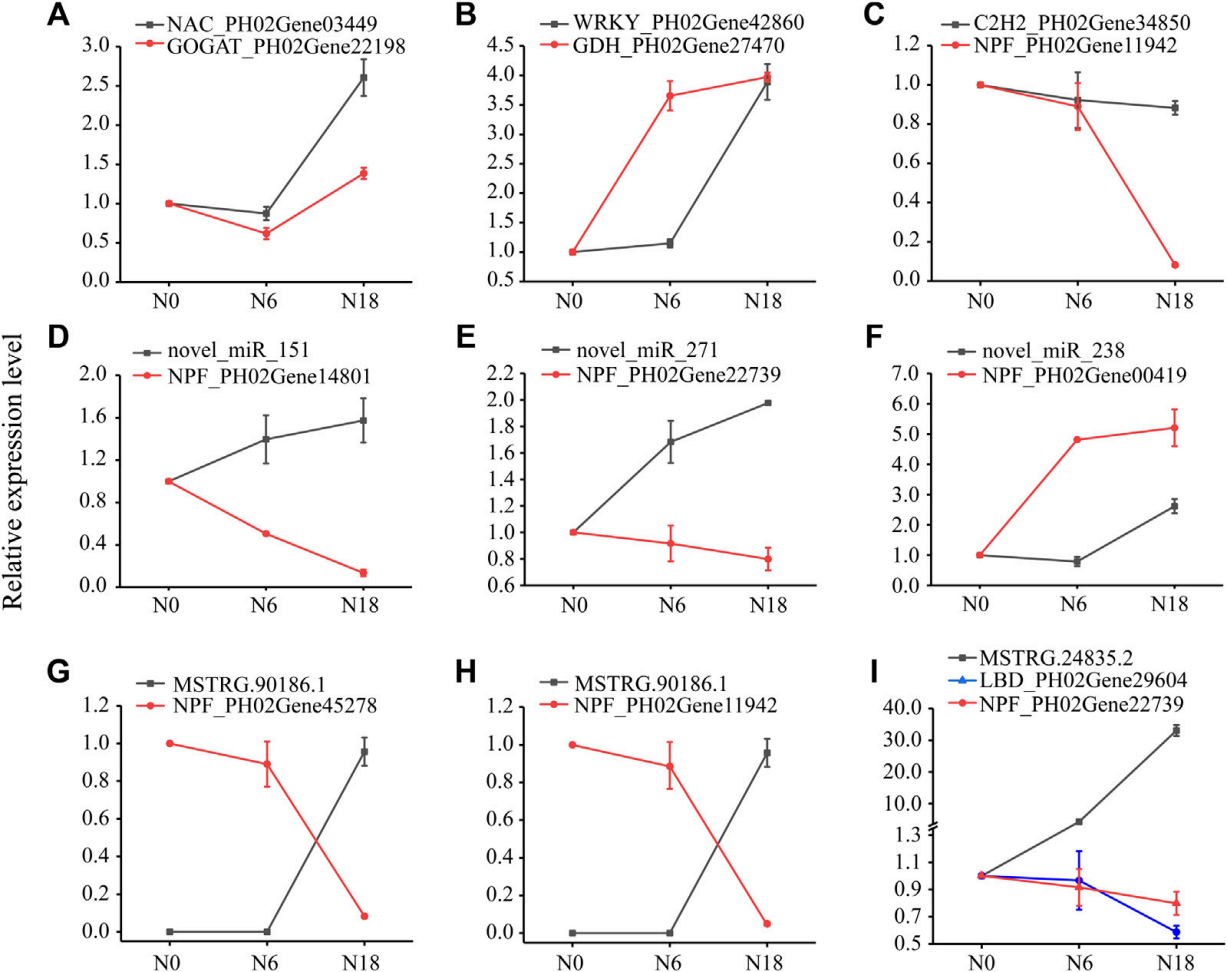
Expression analysis of genes, miRNAs, and lncRNAs using qPCR. Data are means ± SD of three replicates (*n* = 3): **(A)** NAC-GOGAT pair; **(B)** WRKY-GDH pair; **(C)** WRKY-GDH pair; **(D–F)** miRNA-NPF pairs; **(G–H)** lncRNA-NPF pairs; and **(I)** lncRNA-LBD-NPF pairs.

To further explore the potential regulation mechanism of nitrogen metabolism in moso bamboo, an integrated analysis of lncRNA-miRNA-mRNA was performed. Integrating the above results, a regulatory network was constructed, i.e., 17 nitrogen metabolic pathway genes, 15 TFs, 4 miRNAs, and 10 lncRNAs (Figure 7; Supplementary Table S4). To validate the genetic elements in the regulatory network, the qPCR method was used. The results showed that the TFs and their targeted genes had similar expression patterns, such as *NAC*_PH02Gene03449 and *GOGAT*_PH02Gene22198 were both downregulated under N6 and upregulated under N18 (Figure 8A), which was consistent with the transcriptome data. The same situation was found in *WRKY*_PH02Gene42860 and *GDH*_PH02Gene27470, *C2H2*_PH02Gene34850, and *NPF*_PH02Gene11942 (Figures 8B, C). The similar expression patterns further suggested that the enzyme genes were positively regulated by the TFs, which was supported by the binding sites found in the promoter sequences of the enzyme genes (Figure 4C).

*Novel_miR_151* and *novel_miR_271* were upregulated, while their targeted genes (*NPF*_PH02Gene14801 and *NPF*_PH02Gene22739) were downregulated under N6 and N18 when compared with under N0 (Figures 8D, E). In addition, *novel_miR_238* was downregulated and its targeted gene (*NPF*_PH02Gene00419) was upregulated under N6 when compared with under N0, but they were all upregulated under N18 when compared with under N0 (Figure 8F). The qPCR of miRNA-mRNA pairs further validated the results of the high-throughput sequencing. *MSTRG.90186.1* and *MSTRG.24835.2* were upregulated, while their targeted genes (*NPF*_PH02Gene45278, *NPF*_PH02Gene11942, and *LBD*_PH02Gene29604) were downregulated under N6 and N18 when compared with under N0 (Figures 8G–I). Meanwhile, *LBD*_PH02Gene29604 and *NPF*_PH02Gene22739 had similar expression patterns, and putative LBD binding sites were found in the promoter of *NPF*_PH02Gene22739 (Figure 4C), indicating that *MSTRG.24835.2* indirectly regulates NPF genes by targeting TF. These results provided evidence for elucidating the relationship between the genetic elements in the regulatory network of nitrogen metabolism (Figure 7).

## Discussion

### Nitrogen Availability Affected the Phenotype, Physiology, and Biochemistry of Moso Bamboo

Nitrogen has a wide range of effects on plants: nitrogen deficiency stunts plant growth and development, reduces photosynthesis and leaf area, promotes plant senescence, and ultimately decreases plant productivity (Mu et al., 2016). Nitrogen supply has a great effect on biomass production due to its close relationship with leaf area and the longevity of green leaves (Zhong et al., 2019). In this study, nitrogen additions improved chlorophyll content and Pn of moso bamboo seedling leaves, which led to a biomass increase for the whole plants (Figure 1). Similar conclusions were reported in poplar, wheat (*Triticum aestivum*) (Imran et al., 2019), and peanut (*Arachis hypogaea*) (Li et al., 2021). Actually, in our study, the seedlings under N18 treatment had the most chlorophyll accumulation, while the seedlings under N12 had the highest Pn among the treatments (Figure 1). This indicates that a moderate nitrogen level is beneficial to the growth of bamboo seedlings, which is similar to the results for spinach (*Spinacia oleracea*) (Lin et al., 2014). Meanwhile, N6 significantly promoted the biomass of seedlings, but the promoting effect was weakened with increasing nitrogen concentrations when compared with that under N6 (Figure 1), which are consistent with the results of a poplar study (Luo et al., 2013). Therefore, we hypothesized that there is a threshold for nitrogen uptake and assimilation in moso bamboo.

Besides, there are multiple enzymes involved in nitrogen metabolism, in which NR and NiR achieve the two-step reduction. NR is the rate-limiting enzyme, and its activity directly affects the level of nitrogen metabolism in plants (Huarancca Reyes et al., 2018). The activities of NR and NiR in bamboo roots were promoted by nitrogen additions, but they were weakened when the seedlings were exposed to a higher nitrogen condition (Supplementary Figure S2), which agreed with the NR activity in rice exposed to different nitrogen conditions (Gao et al., 2019). The activity of GS was the highest under N0, while GOGAT activity significantly increased after nitrogen additions (Supplementary Figure S2). This may be explained by the feedback regulation between the GS/GOGAT pathway and the posttranscriptional modulation of GS (Ferreira et al., 2019). The activity of GDH was also inhibited by nitrogen additions (Supplementary Figure S2), and the reason may be that GDH acted as a link between carbon and nitrogen metabolism, which was influenced by multiple factors (The et al., 2020). In consequence, nitrogen metabolism of bamboo was greatly affected by the fluctuating nitrogen conditions. Further experiments are needed to explain the variations of the enzyme activities in the nitrogen metabolism of moso bamboo.

### Nitrogen Additions Affected the Expression of Genes Involved in Nitrogen Metabolism

Nitrogen is absorbed and assimilated by nitrogen metabolic pathway genes, and the most immediate effect caused by nitrogen additions is the transcript change of the genes involved in this pathway (Vidal et al., 2020). According to previous studies, genes encoding NRT1 and NRT2 were rapidly induced by nitrate within hours or days in *Arabidopsis* (Wang et al., 2000). Similarly, the expression of several *NPF* genes was induced in the presence of nitrate, such as *OsNPF7.2* (Hu et al., 2016) and *OsNRT1.1* (Hu et al., 2015). In this study, 10 members of the NPF family were induced by nitrogen additions (Figure 3), 32 members were suppressed by nitrogen absence, and 4 members were inhibited by both nitrogen absence and addition (Figure 3). This may be related to the functional diversity of NPF family members. Some NPF members transform to low- or high-affinity transporters by phosphorylation to deal with the fluctuant nitrogen environment, such as AtNRT1.1 in *Arabidopsis* and MtNRT1.3 in *Medicago* (*Medicago truncatula*) (Pellizzaro et al., 2014; Wang et al., 2020). It has been shown that *NRT2.1* is induced upon initial nitrate supply and repressed by nitrogen metabolites or high nitrate provision (Muños et al., 2004). In addition, NRT2 could not independently complete the transport of nitrate, and they needed the assistance of NRT3 (Jacquot et al., 2020). In this study, three members of NRT2 were repressed, and one was induced by nitrogen additions (Figure 3). Interestingly, the expression pattern of two *NRT3* was similar to that of *NRT2*_PH02Gene31118 but was opposite to that of the other *NRT2* (Figure 3). It is speculated that the transport pattern of NRT2 with the partner of NRT3 also existed in moso bamboo, and NRT2 may have dual-affinity transport activity.

NRs play a vital role in nitrogen acquisition, and their mRNAs can be rapidly accumulated in response to nitrate in *Arabidopsis* (Wang et al., 2000). Besides, genes encoding NiR are also induced over a similar range of nitrate concentrations in maize (Trevisan et al., 2011). In bamboo, five NR and two NiR genes were upregulated by nitrogen additions (Figure 3), and the activities of NR and NiR were enhanced (Supplementary Figure S2), illustrating that the reduction of NR and NiR as well as nitrogen metabolism were strengthened after nitrate addition. With nitrate addition, genes encoding specific isoforms of GS and GOGAT involved in ammonium assimilation were upregulated or downregulated due to their multiple forms in different compartments of the cell (Gaufichon et al., 2016). In *Arabidopsis*, *GLN1;2* coding GS1 was found to be preferentially expressed in the roots and induced by nitrate reduction, while *GLN1;2* and *GLN1;3* did not show marked induction due to a low affinity for ammonium (Konishi et al., 2017). *OsGS1;1* and *OsGS1;2* were expressed in all organs and showed a reciprocal response to ammonium supply in rice roots (Tabuchi et al., 2007). In bamboo, the transcripts of two GS genes were found to be more abundant and had similar expression patterns with most nitrogen metabolic pathway genes after nitrate addition, indicating that they were nitrate induced (Figure 3). One GOGAT gene was induced and another was inhibited by nitrate addition. Meanwhile two GDH genes were induced and one was inhibited by nitrate addition (Figure 3), indicating the expression diversity of different members in the same family. These results are in accordance with the previous report of GDH genes in tobacco (*Nicotiana tabacum*) (Tercé-Laforgue et al., 2015). In conclusion, nitrogen additions affected the expression of most genes of the nitrogen metabolic pathway, either induced or inhibited, which helped bamboo cope with the fluctuant nitrogen.

### TFs and Noncoding RNAs Participated in Nitrogen Metabolism by Regulating Transporter and Enzyme Genes

To date, more than 40 TFs of several families have been identified to be involved in nitrate transport, nitrate reduction, and nitrate assimilation through regulating the expression of genes in nitrogen metabolism (Maeda et al., 2018). Previous studies have shown that *MYB61* was induced by nitrogen and showed a similar expression pattern with nitrogen homeostasis genes (*NRT1.1* and *NIA1*), and MYB bound the promoter of *NRT*, *NiR*, and *GS* (Imamura et al., 2009; Gao et al., 2020). AtNLP6/7 was induced by a nitrate signal, and then bound the promoter of *NRT2.1* and *NIR1* in the presence of nitrate (Liu et al., 2018). LBD37-39 involved in nitrogen metabolism by signaling nitrogen availability, leading to repression of anthocyanin metabolic pathway genes and feedback repression of *NIA* or *NRT2* (Rubin et al., 2009). In this study, 431 TFs belonging to 23 families were identified to participate in nitrogen metabolism, such as MYB, NAC, LBD, WRKY, and C2H2 (Figure 4). LBD was predicted to regulate *NPF*, and MYB putatively regulated *NPF*s and *GDH*, due to the predicted binding site found in the promoters as well as the positive correlation of their expression levels (Figure 4). In other words, many TFs were involved in bamboo nitrogen metabolism and might play important roles.

Recent research has focused on miRNAs and lncRNAs regulating the expression of specific genes related to nitrogen metabolism (Vidal et al., 2010; Liu et al., 2019). In *Arabidopsis*, *miR827* was involved in the translational repression of NLA, which directed the ubiquitination of *NRT1.7* and regulated the remobilization of nitrate (Liu et al., 2017). The transcript level assays showed that *miR169o* oppositely regulated *NRT2* under different nitrogen conditions in rice (Yu et al., 2018). In addition, *miR166*, *miR169*, *miR408*, and *miR528* displayed a crucial step in integrating nitrate signals into developmental changes in maize roots upon nitrate shortage (Trevisan et al., 2012). In this study, 383 miRNAs were identified to participate in nitrogen metabolism of moso bamboo, among which four miRNAs targeted three *NPF*s, which was supported by the expression profiles based on the transcriptome data (Figure 5). Besides, miRNAs are also involved in the nitrogen metabolism by targeting TFs, which in turn targeted nitrogen metabolic pathway genes. NFYA was targeted by *miR169* while it targeted *NRT1.1* and *NRT2.1* in response to nitrogen changes (Zhao et al., 2011). Also, *miR164* was upregulated under nitrogen starvation and resulted in downregulating the expression of *NAC* (Guo et al., 2005). In this study, 29 DEMs were identified to regulate TFs, such as *osa_miR535_3p* targeted *NF-Y*, 7 DEMs targeted *NAC*, and *novel_miR260* targeted *SPL* (Supplementary Figure S3), indicating they play important roles in nitrogen metabolism of moso bamboo.

Although available reports indicated that lncRNAs served as essential regulators of various plant biological processes, studies on lncRNAs involved in nitrogen metabolism are limited. lncRNA *T5120* played an important role in nitrate uptake and nitrate assimilation by regulating the expression of *NTR1.1* in *Arabidopsis*, which resulted in improved crop biomass (Liu et al., 2019). Besides, 2,588 lncRNAs and 388 lncRNAs were identified as participating in nitrogen metabolism in rice and poplar, respectively (Chen et al., 2016; Shin et al., 2018). In this study, 10 lncRNAs were identified, which were speculated to participate in nitrogen metabolism by targeting *NPF*s and *GDH*s directly or indirectly through TFs (Figure 6). In total, several miRNAs and lncRNAs involved in nitrogen metabolism of moso bamboo were identified. Further experiments were still needed to verify their functions.

The process of nitrogen metabolism involves complex gene regulation. Based on a series of comprehensive analyses, a multilevel regulatory network, the lncRNA-miRNA-mRNA module of nitrogen metabolism, was successfully established and initially validated in bamboo (Figures 7, 8). The regulatory network helps illustrate the mechanism of nitrogen metabolism in moso bamboo. By manipulating a single TF or lncRNA, targeting multiple genes in the nitrogen metabolism pathway could be realized at the same time, which has a greater effect than overexpression of a single structural gene. The regulatory network provides a more efficient way of manipulating gene expression to improve the nitrogen metabolism of bamboo under different nitrogen supplies.

## Conclusion

Appropriate nitrogen addition improved nitrogen metabolism and promoted growth of roots, and as a result, Pn and biomass of the seedlings increased. Based on the combination of multiple RNA-seq analyses of the expression profiles of mRNAs, miRNAs, and lncRNAs in bamboo roots under different nitrogen additions, a regulatory network of nitrogen metabolism was constructed, such as 17 nitrogen metabolic pathway genes, 15 TFs, 4 miRNAs, and 10 lncRNAs. The lncRNA-miRNA-mRNA network reveals the regulation mechanism of nitrogen metabolism in moso bamboo and provides candidate gene resources for improving the ability of bamboo to adapt to a fluctuating nitrogen environment.

## Data Availability

The original contributions presented in the study are publicly available. This data can be found here https://www.ncbi.nlm.nih.gov/ PRJNA797724 AND PRJNA797734.
