# Supplementary material for: An Integrated Regulatory Network of mRNAs, microRNAs, and lncRNAs Involved in Nitrogen Metabolism of Moso Bamboo
Source: Front Genet. 2022 May 16;13:854346. doi: 10.3389/fgene.2022.854346 (PMC9149284; doi:10.3389/fgene.2022.854346)
Supplement: Supplementary file 3 [file Presentation1.PPTX]

## Slide 1
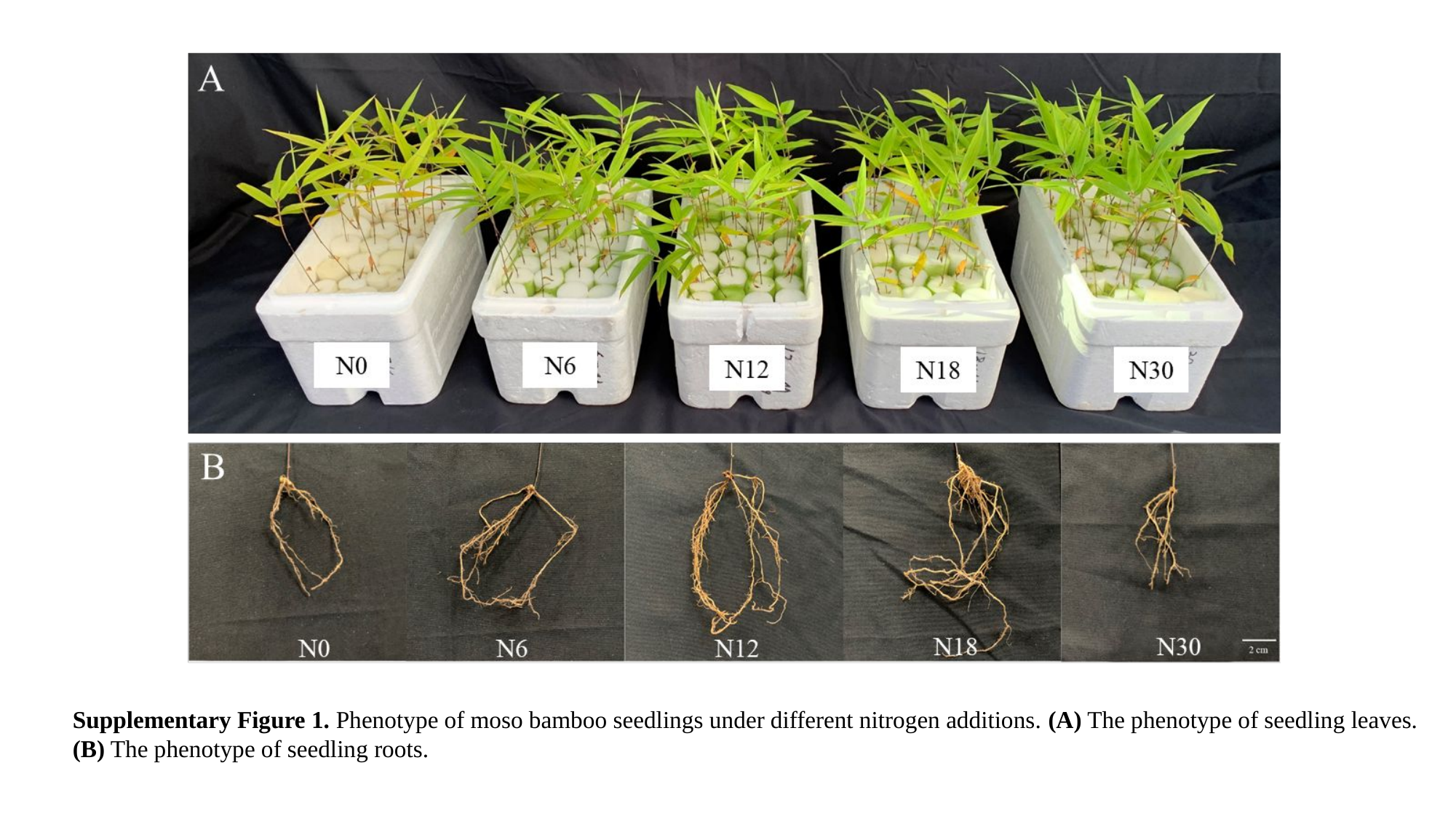

Supplementary Figure 1. Phenotype of moso bamboo seedlings under different nitrogen additions. (A) The phenotype of seedling leaves. (B) The phenotype of seedling roots.

## Slide 2
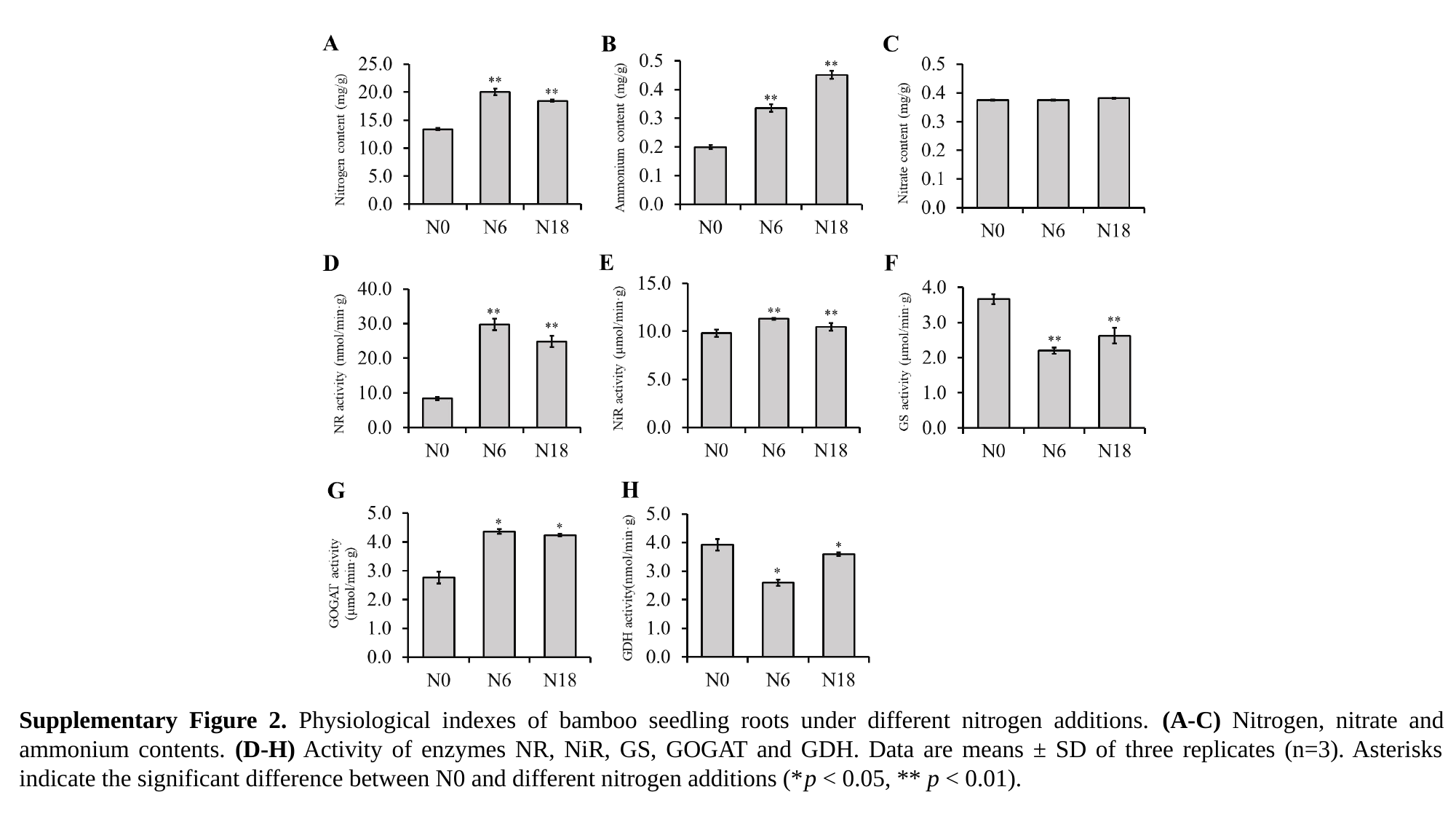

Supplementary Figure 2. Physiological indexes of bamboo seedling roots under different nitrogen additions. (A-C) Nitrogen, nitrate and ammonium contents. (D-H) Activity of enzymes NR, NiR, GS, GOGAT and GDH. Data are means ± SD of three replicates (n=3). Asterisks indicate the significant difference between N0 and different nitrogen additions (*p < 0.05, ** p < 0.01).

## Slide 3
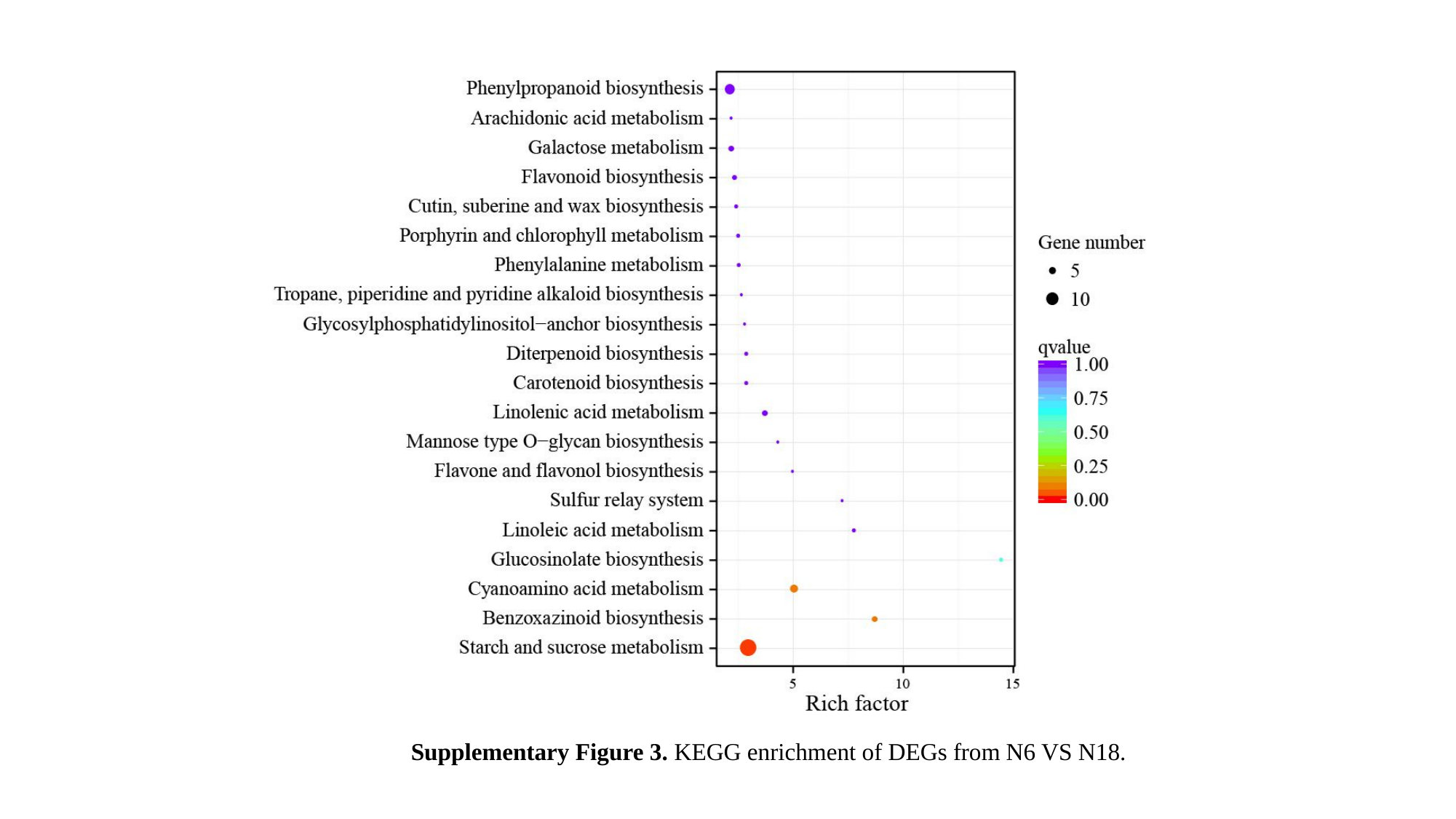

Supplementary Figure 3. KEGG enrichment of DEGs from N6 VS N18.

## Slide 4
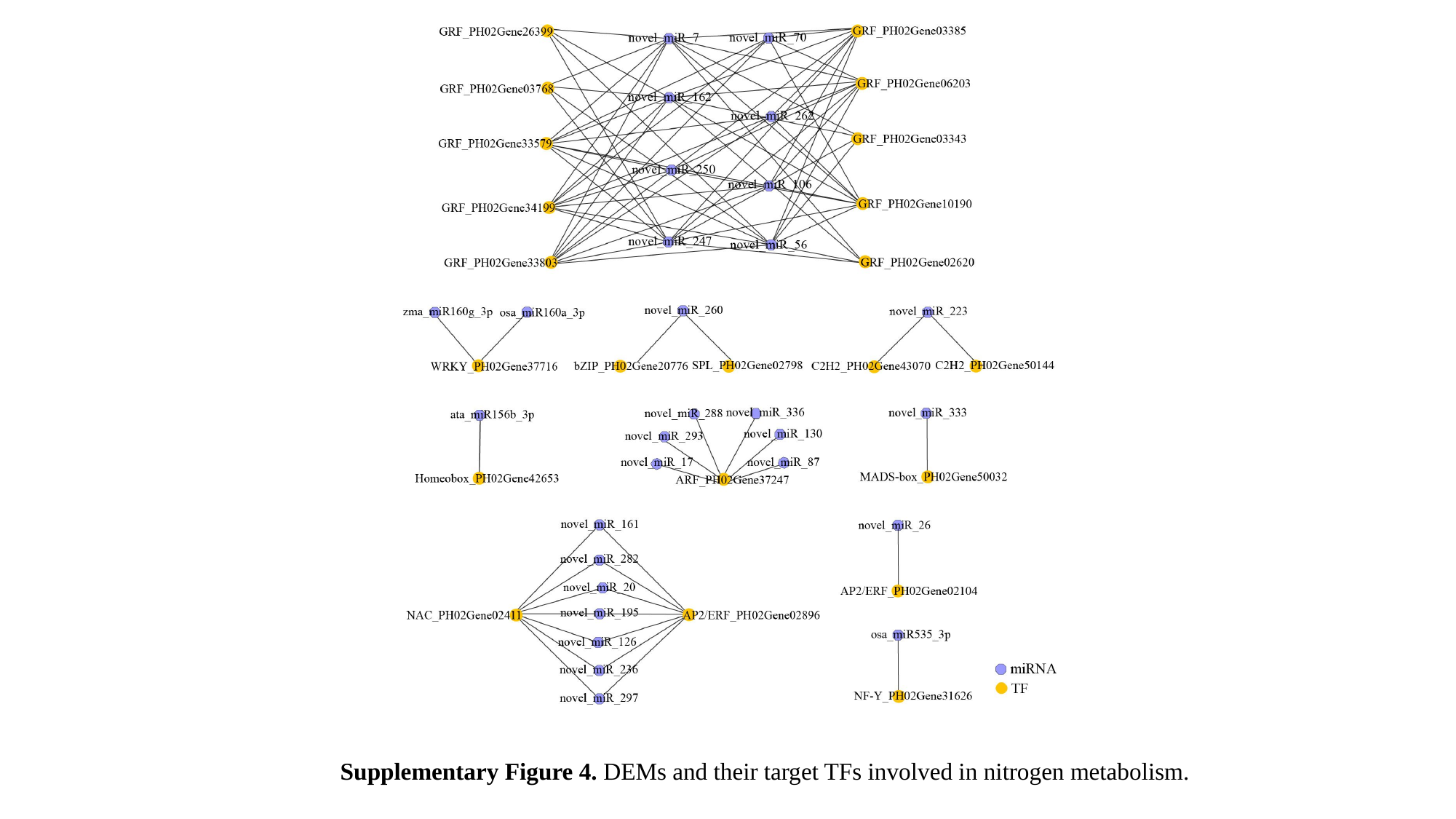

Supplementary Figure 4. DEMs and their target TFs involved in nitrogen metabolism.
